# Parental Perception of Children Affected by Amelogenesis Imperfecta (AI) and Dentinogenesis Imperfecta (DI): A Qualitative Study

**DOI:** 10.3390/dj6040065

**Published:** 2018-11-17

**Authors:** Areej Alqadi, Anne C. O’Connell

**Affiliations:** Division of Public and Child Dental Health, Dublin Dental University Hospital, Dublin, Ireland; alqadia@tcd.ie

**Keywords:** dental anomalies, parents, children, dentist, aesthetics, primary dentition, amelogenesis Imperfecta, dentinogenesis imperfecta, enamel defects, psychosocial impacts

## Abstract

This qualitative study was conducted to explore parental attitudes and values regarding aesthetics and treatment needs of children in primary dentition affected by AI and DI. A purposive sample of parents of young children attended two focus groups: mothers (n = 7) and fathers (n = 6). A topic guide with open-ended questions was formulated and standardised photographs showing primary teeth affected by varying severity of AI/DI and photographs of different aesthetic treatments were utilised to stimulate discussion. Data was audio-recorded and transcribed verbatim. A cross-sectional thematic analysis was performed which identified six main themes; the impact on affected children, the impact on parents, the life course of the disease, coping mechanisms, treatment need, and experience of treatment. Parents believed that young children were aware of their altered dental appearance. A feeling of guilt was evident among fathers affected by the same condition. Most parents sought dental treatment before starting school due to worries of bullying at school. Parents appeared to rely solely on the professional advice of the paediatric dentist in making all treatment related decisions. The personal experience of parents affected by AI/DI played a pivotal role in parent’s judgements of their children’s teeth and perceived need for treatment.

## 1. Introduction

Amelogenesis Imperfecta (AI) is a hereditary developmental disorder which affects the structure and clinical appearance of enamel of all or nearly all the teeth in a more or less equal manner [1] and affects both dentitions. The prevalence has been reported to range from 1.4:1000 to 1:16,000 depending on the population studied [2]. Depending on the stage of enamel formation that is affected, AI affected teeth can present as hypoplastic (thin enamel, surface pitting or vertical grooving), hypomineralised (soft enamel), or hypomature (Mottled/opaque/altered enamel translucency). Both hypomineralisation and hypoplastic defects can coexist in the same patient and even the same tooth [1]. AI affected teeth are often discoloured, creating aesthetic problems in addition to sensitivity, tooth wear, loss of vertical dimension of occlusion, increased calculus formation, and the need for lifelong dental care. 

Dentinogenesis imperfecta (DI) is the most common type of hereditary developmental disorders of dentine with an estimated incidence ranging between 1:6000 and 1:8000 [2]. DI affected primary and permanent teeth vary in colour and can exhibit grey, blue, yellow, brown, and opalescent discolouration. Tooth discolouration tends to be more marked in the primary dentition due to thinner primary teeth enamel. Additionally, DI-affected teeth may become altered in shape due to wear and attrition. 

The shape, size, and colour of teeth contributes to dentofacial appearance. Alterations in any one of these attributes may affect facial attractiveness and influence how individuals are perceived by society and how they perceive themselves. The impact of dentofacial attractiveness on social judgements of personal characteristics and social interactions of children [3,4,5] and adults [3,5,6] has been well documented. People are more likely to make negative judgments of social and intellectual competence based on altered dental appearance. Several studies with variable methodologies have investigated the aesthetic perception and psychosocial impact of developmental enamel defects affecting the anterior permanent teeth with a focus on adolescents and young adults [7,8,9,10]. Subjects with enamel defects were perceived as being lazy and not caring about appearance [10] and reported being worried and embarrassed about their dental appearance [11].

A cross-sectional controlled study by Coffield et al. demonstrated a significant psychosocial impact of AI on affected adults in terms of social interaction, anxiety, self-image, self-esteem, and self-perceived quality of life. Affected individuals were found to be unhappier with their dental appearance with 93% reported being teased about their dental appearance. Moreover, affected individuals had higher levels of social avoidance and distress compared to other unaffected family members who served as controls [12]. Similarly, a negative psychosocial impact on AI affected adults and adolescents was reported in Ireland, Sweden and UK, however, the self-esteem was not significantly affected [13,14,15].

Collectively, recent studies indicate the psychosocial impact of AI on affected adolescents and adults. Surprisingly, the psychosocial impact of Dentinogenesis Imperfecta on affected individuals and their perceptions of the condition have never been investigated. No study has assessed the perception of parents whose children have primary teeth affected by AI/DI, particularly where many of these parents may be affected themselves or have a family history of these dental anomalies. There is a dearth of both quantitative and qualitative research which has explored the psychosocial impact of AI/DI on young children and their parents 

Parents often present to the dentist with concerns about the appearance of their child’s affected teeth. A better understanding of both mothers’ and fathers’ attitudes, beliefs, values, and expectations regarding aesthetics and treatment need is required to facilitate communication with parents. The aim of this study was to investigate and explore parental attitudes, beliefs, values, and expectations regarding aesthetics and treatment need of children in primary dentition affected by AI and DI.

## 2. Materials and Methods

Ethical approval was granted by the Ethics Committee of the Faculty of Health Sciences in Trinity College, Dublin (reference number: 160603). The main researcher (AA) attended a training course in qualitative research methodology before conducting the study. This study took place in the Dublin Dental University Hospital (DDUH) which is a national referral centre for children with developmental dental anomalies. All patients diagnosed with AI or DI are recorded on a specific data base since 2002 (Special Dental Need Database (SPNS)). Dental treatment for patients on this database is provided at no cost to the patient. The electronic patient management system (SALUD) and the existing Special Dental Needs database (SPNS) held in the DDUH were used to identify participants who fulfil the inclusion criteria.

The inclusion criteria were:

- Parents of children 10 years old or under referred to DDUH;
- Agreed diagnosis of AI/DI affecting their primary dentition;
- Able to speak English; and
- Able to provide consent.

Eligible parents were approached through the gatekeeper (AB) and those who agreed to participate were divided into two separate focus groups; one including mothers and one including fathers as this was assumed to facilitate disclosure and help in shaping effective group dynamics. Purposive sampling was applied to include parents of boys and girls affected by either AI or DI with a wide range of age (above 35 and below 35 years of age), and where different levels of dental intervention has been provided for their affected children. In addition, purposive sampling was utilised to ensure that each focus group included parents affected by AI/DI.

In qualitative research it is often difficult to know exactly how many participants are required in advance, however, the focus groups were planned to comprise of 6–12 participants as is recommended to provide appropriate group dynamics [16]. The total number of focus groups was determined by theoretical saturation. Theoretical saturation is the point in data collection that is achieved when no more new themes emerge from the newly-collected data [16,17].

A topic guide was formulated to elaborate on the aspects the researchers are interested in. The guide consisted of open-ended questions that explored five key areas including:

1. Aesthetics and attractiveness;
2. Impact on the child;
3. Impact on parents;
4. Treatment need; and
5. Influence of dental interventions.

The questions were informed by the literature regarding AI and DI as well as the literature regarding parental perception of dentofacial anomalies and clinical experience. The topic guide was formulated by the research team, was revised by an independent experienced qualitative researcher (AD) and was updated after each focus group. Eleven standardised photographs of a smile showing teeth affected by varying severity of AI/DI and photographs of different aesthetic treatments (composite strip crowns, zirconia crowns, a mix of anterior composite/zirconia crowns, and posterior stainless steel crowns) were utilised to stimulate the discussion and parents were encouraged to express their attitudes and feelings toward these photographs.

The focus groups were guided by an independent moderator (AD), who had extensive experience of in depth qualitative interviews and focus groups moderation. The main researcher (AA) provided the moderator with the topic guide, attended the focus group session and was the note keeper. The researcher indicated to the moderator when certain issues needed further probing. Field notes were prepared before and after the focus group meeting and were used to record observations, non-verbal gestures, and other significant events.

In order to limit the influence of the researcher and research process on the collected data, the experienced and independent moderator raised focus group questions based on the prepared topic guide in a neutral, non-leading way, the moderator and the researcher were unknown to the participants, the moderator introduced herself as a person with no experience with AI and DI, dental jargon was avoided, and the participants were assured there was no right or wrong answers. In addition, the location for focus group meeting was a quiet meeting room in the Dublin Dental University Hospital away from the dental working area to ensure privacy and provide a less stressful and non-clinical environment.

**Figure 1:**
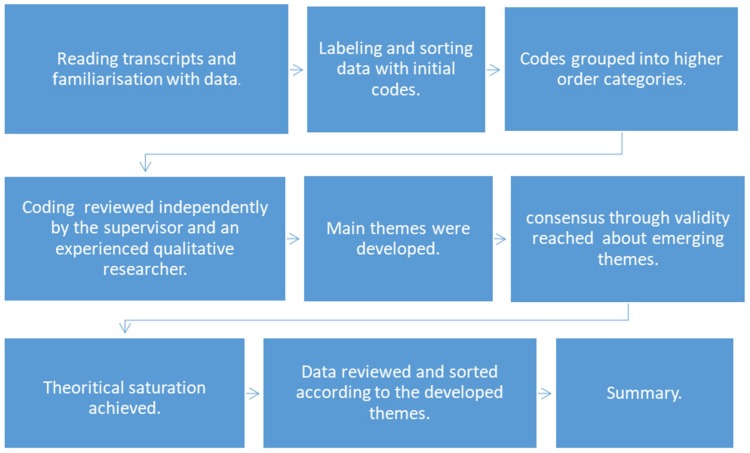
Steps of thematic analysis.

Data were audio-recorded on two separate recording systems to guarantee no loss of data. The records were transcribed into Microsoft Word (2013, Microsoft Corporation, WA, USA,) and the participants were given different names in the transcribed data to ensure full confidentiality. The transcripts were then uploaded to a qualitative data analysis software (MAXQDA standard, VERBI Software, Berlin, Germany). The software was used by the researcher for thematic analysis by systematically evaluating and interpreting textual data. A cross-sectional thematic analysis approach was followed in analysing the data of all focus groups. It is a flexible qualitative research analysis approach for identifying and reporting patterns of meaning across the whole dataset, thus providing a rich, detailed interpretive, yet complex, account of data [18]. It involved multiple steps, as illustrated in Figure 1.

Rigour and trustworthiness were established by adherence to quality guidelines for the qualitative research conduct [19], revisiting issues and requesting clarification during the course of the focus groups and by triangulating interpretations among the research team. Members of the research team and an experienced qualitative researcher (BD) who was not involved in data collection reviewed the data independently before the main themes were agreed to ensure both consistency and comprehensiveness in coding and data analysis and to limit any researcher bias.

## 3. Results

A total of fifty six eligible parents were contacted through the appointed gatekeeper, eighteen parents agreed to participate (eight fathers and ten mothers). Two focus groups were conducted involving a total of thirteen parents of affected children. Seven mothers participated in one focus group held on 2 March 2017 and six fathers participated in the other focus group held on 16 February 2017. Both meetings were arranged in the afternoon based on participant’s preference. The fathers’ focus group lasted for one hour and fifty minutes and the mothers’ focus group lasted for one hour and twenty-four minutes. The number of transcript pages analysed were 129 and 178 for the fathers’ and mothers’ focus groups, respectively. Data review and analysis were done in conjunction with data collection and the quality of data obtained from two focus groups was rich and experiential and was judged to be sufficient to reach thematic saturation. The demographic characteristics of participants are summarized in Table 1 and Table 2.

Cross-sectional analysis identified six main themes; the life course of the disease, the impact on affected children, the impact on parents, coping mechanisms, treatment need, and experience of treatment.

### 3.1. The Life Course of the Disease

Most parents started to notice an altered appearance of their children’s teeth as soon as the first primary teeth were erupting. 

***CC**: “Like you’d see, the minute you’d see it come through the skin, you can see it. You know straight away.” (Father of a child affected by DI)*

Regardless of the family history of developmental dental defects, parents were keen to get dental advice as soon as they started to notice the defect in their children’s teeth. Some were referred to the DDUH through their own dentists while others used Google search to get to the DDUH and arranged for their children’s dental assessment by themselves. Affected parents who were themselves patients of the DDUH found their way to the paediatric service in DDUH at an early time point. The age of the child when parents sought referral varied but most referrals occurred between one to three years of age. Those who sought referral later had been attending their family dentist for regular check-ups.

In general, parents were reluctant to accept the myth “that baby teeth are not important and will fall out anyway” and exhibited a persistent attitude to get expert assessment, advice, and treatment.

***D**: “But it is that old-fashioned thing of, ah, the baby teeth, don’t worry about them.” (Mother of a child affected by DI)*

***C**: “They have the baby teeth till they’re seven or eight years of age. Of course they’re important, you know.” (Mother of a girl affected by DI.)*

A number of parents postulated that their children’s teeth worsened over time. In contrast, other parents believed that the permanent teeth are usually less affected. A minority of participants recalled that the diagnosis for their children was not made until their early mixed dentition.

A recurrent theme in the focus groups was a sense of acknowledgement of the lifelong dental treatment need of affected teeth, mixed with uncertainty about future treatment needs. 

***CC**: “Because it is a lot of years, it’s not just one. The parent has to be prepared like it’s not going to be a short-term fix, like it’s a lot of years and travelling, money and operations, like they have to be prepared for everything then.” (Father of a child affected by DI.)*

A variety of perspectives were expressed in relation to that including; focusing on the present, preparing the child to anticipate progressive and continuous dental treatment and investing family savings.

### 3.2. The Impact on Affected Children

Parents, on the whole, demonstrated a belief of an increased social expectation of white, well-aligned teeth and a social perception of white teeth as an indication of personal hygiene. It was suggested that young children are influenced by this social phenomenon and so they are expecting normal primary teeth to be pearly white and well-shaped teeth. In fact parents described their children’s teeth as “not as white as other children teeth”, “discoloured”, “yellow looking”, “smaller”, “thinner”, “not solid looking”, “jagged and pointed”, “spiky”, “rough”, “not healthy looking”, and “see-through—if you shone a light you could see nearly through the tooth”.

***JMN**: “There is an expectation now to be personal hygiene, you know, like we’re not going out the back garden to the toilet so yeah. So there’s an expectation.” (Father of a child affected by AI.)*

***JMC**: “And it can kind of hit you a little bit more when your son at three years of age turns round and says “Dad, why are your teeth green?” because he’s expecting white.” (Father of a child affected by AI.)*

Parents believed that this altered dental appearance can make children susceptible to negative social reactions and referred to their own experience when they were children and their children’s experience to support their beliefs. These accounts focused on being seen and/or treated differently and varied between staring, questioning teeth appearance, questioning teeth brushing, assuming overconsumption of sugary food and bullying at school.

***A**: “kids in school, they used to say why are your teeth so small or, yellow, and her friend said no matter how long she brushes them, they’ll always be yellow.” (Mother of a child affected by AI)*

There was a general agreement between parents that young children at preschool and primary school age could notice affected teeth and might show innate unfavourable responses.

***CS**: “I don’t have it, so like we never really kind of mentioned it to my son, and he’s in senior infants now and I suppose it’s been mentioned. He is come back from school a couple of times and said oh, some of the boys and girls were saying my teeth are a bit yellow.” (Father of a child affected by AI.)*

***JMA**: “<Oh, definitely, yeah, young children can pick it up more so than adults. Like children have no filter” (Father of a child affected by DI.)*

Parents believed that their children would be aware of the altered dental appearance before three years of age and were concerned about the impact of that on the child’s confidence and self-esteem.

***CL**: “I’m actually telling, my two-year-old already knows her teeth are different to her mam’s teeth and she’ll say it like, and she’ll say things like oh, why do I have cheese on my teeth, is usually what she’s saying. She barely would know what’s in the world but she can already distinguish that.” (Father of a child affected by AI)*

***H**: “She’s two and four months, and we don’t really kind of make a big deal about teeth when we talk, but if you want to talk about something, talk about it, and she is always on about teeth.” (Mother of a child affected by AI)*

Opinions of parents differed as to whether the child’s gender will influence the extent of psychosocial impact. Some thought that girls are more affected, others argued that boys are becoming more concerned about their own appearance and so might be more or equally affected.

From a functional perspective, the majority of parents expressed their concerns in relation to the affected teeth being friable and vulnerable to breakdown and wear. There was a general belief among parents of affected children that they are more conscious about the food intake of their child when compared to parents of unaffected children and consequently reported that their diet was healthier.

Interestingly, affected parents relied solely on their own experiences to assume that children’s teeth were sensitive and friable even when they were not.

***E**: “For a long time I wouldn’t let him eat ice cream because I couldn’t touch ice cream because of the cold, I was very sensitive to the cold, and then he was in the crèche and they had this birthday party thing and they gave out ice cream and I had put on the thing don’t give him ice cream, so he comes back and he says Mammy, the ice cream, it’s lovely, no he didn’t have the sensitivity that I had.” (Mother of a child affected by AI, Mother is affected.)*

Parents avoided offering their children certain foods and drinks (ice cream, fizzy drinks, toffies, jellies, hard food) due to sensitivity and breakdown concerns.

### 3.3. The Impact on Parents

Four key emotions of parents about having an affected child arose during the discussions; sadness, fear of bullying and negative social reactions, worry about associated impact on the child and the lifelong treatment need and a feeling of guilt.

***C**: “I haven’t ever heard of it in my life, I was roaring and crying then going to her, she’s gonna be teased.” (Mother of a girl affected by DI.)*

***H**: “Oh, my heart breaks, because I’m looking at them, because it’s something different like, I think anything different, you don’t want your kid to be bullied.” (Mother of a child affected by AI)*

There was three main ways in which the feeling of guilt manifested within the experiences of affected parents. Firstly, the inheritance of a developmental dental defect is a reason to feel responsible for all the difficulties the child is expected to go through.

***CL**: “You still feel a little bit responsible because I guess it’s your genes and not your partner’s and then some of it’s also just a bit of cursing your luck as well.” (Father of a child affected by AI)*

The second manifestation relates more specifically to seeking dental treatment and the difficulties and complications that might occur in association with early dental treatment.

***H**: “My little fella then he got one strep throat after another, and of course I put it all back to getting the teeth done, and I did feel guilty” (Mother of a child affected by AI)*

The third manifestation was the feeling of guilt if dental treatment was not sought by parents.

***JMN**: “I’d feel guilty if I wasn’t taking steps to help him, that’s guilt” (Father of a child affected by AI—father is affected)*

This feeling of guilt appeared more prominent in the father’s focus group. Conflicting views, however, were expressed about the degree of this guilt, with some expressing a very large feeling of guilt that extends to affected grandparents, and others denying any feeling of guilt based on the personal lack of control on genetic inheritance and their own positive experience with dental treatment.

### 3.4. Coping

A number of strategies were employed by parents to cope with having children affected by AI/DI. Within this theme, the following subthemes were evident from the data:

- Acknowledgment and explanation: Many parents used the term “special teeth” to describe the condition to their children.
- Positive parents’ comments on appearance.
- Promise of future definitive dental treatment.
- Photoshop of family photos.
- Seeking dental treatment.
- Diet modification.
- Feeling grateful for not having other major systemic diseases.

### 3.5. Treatment Need

Most parents sought dental treatment of their children around the age of two to three years and were concerned about getting dental treatment before their children started primary school.

Several reasons for seeking dental treatment was advocated by parents including mainly psychological reasons attributed to appearance as well as other functional reasons. The main reasons proposed by parents were: 

1. Child protection.

There was a general expectation that early dental treatment will protect children from ‘feeling different’ at young age and, thus, protect their self-esteem and confidence as they grow up. Starting primary school was referred to as a critical period within the life course of the disease as most parents believed that the children’s awareness of a difference, as well as other children’s innate negative responses to the appearance, increases incredibly around this age. Concerns and fears about bullying at school as a main motivating factor for treatment were repetitively referred to by parents in both focus groups.

***JMA**: “You’re trying to protect the child, you don’t want them to be slagged, and I think that’s why we went for the option when she was so young to get it done, because before she starts school then, to get it done because she’ll know no different.” (Father of a child affected by DI.)*

Interestingly, parents believed that young children lack the capability to respond to other people’s comments at this age, thus, the dental treatment can provide them a shield of protection until they get older and be more capable to respond to people’s comments. Parents wanted to ‘make life easier on the child’ (in the words of participants). Apparently, affected parents’ concerns of bullying were attributed to their own experience of bullying during childhood. However, the affected parents’ experiences with dental treatment significantly influenced their motivation for early dental treatment of their children. Those who had positive dental experiences showed more enthusiastic attitudes toward dental treatment.

2. Achieving normality.

***JMA**: “I just wanted what was best for the child. Just as long as her teeth were happy and they’re, not happy but healthy and looked half-normal.” (Father of a child affected by AI.)*

3. Maintaining structure and function.

***D**: “to keep them in place for longer.” (Mother of a child affected by DI)*

Issues related to sensitivity and dietary problems as motivating factors for treatment were not particularly prominent in the focus group data.

### 3.6. Experience of Treatment

Expert opinion and advice were identified as particularly critical factors in treatment decisions. This view was held by almost all parents. Most parents relied on the paediatric dentist who is the expert from parents’ perspective as the main source of information and the decision-maker on most treatment-related decisions particularly when choosing between different pharmacological behavioural interventions. 

***D**: “I sound like I’m probably disinterested in my children’s teeth, I haven’t Googled anything. I never Googled it. I just do everything Dr (A) says I go with and if she advises to do X, Y or Z.” (Mother of a child affected by DI)*

***H**: “I’m always going to take the professional’s advice, she’s not gonna push you into a theatre if it’s not necessary.” (Mother of a child affected by AI)*

***JMA**: “Well I think in our case Doctor (A) put it to us that this was the best option, and it was the best time to have it done so we knew nothing about it so we trusted her.” (Father of a child affected by AI.)*

Parents who had an experience of reading about it from different sources were frustrated with the type of information they obtained in terms of limited amount and ‘depressing’ quality.

Not surprising, all parents wanted beautiful aesthetic smiles of their children and were completely against getting silver (metal) teeth unless recommended by the clinician and/or when they were not visible when the child smiles.

Parents talked about their experiences in deciding on dental treatment under general anaesthesia (GA). They highlighted the difficulties they have faced at several stages, including making the decision for treatment, the time of induction, during and after the operation. These difficulties were particularly prominent in the mothers’ focus group.

The data revealed that most children had no experience with general anaesthesia before receiving this dental treatment and this added to the difficulty of making the treatment decision. In addition, parents referred to being blamed by the general society for putting the child through the risks of general anaesthesia only for dental treatment of primary teeth that will be lost eventually.

***CL**: “Giving a three-year-old a general anaesthetic is not something <you actually do lightly>” (Father of a child affected by AI)*

***F**: “And people were saying why are you putting the child through that? They thought I was a really bad mother, why are you putting the child through that when it’s only baby teeth and he’s going to lose them?” (Mother of a child affected by AI)*

Overall, the participants demonstrated general satisfaction with early dental intervention of their children’s primary teeth. Apparently, this satisfaction was three-fold, the positive psychosocial impact on the child, the improved aesthetic appearance, and the feeling of smoothness of the teeth.

***CL**: “I can see the difference in my son, he’s not self-conscious, he is an extremely confident little man and, I think by the time his front main ones come down, I think if someone comes round to him and says something, I think he’ll turn back around and say well, do you know what, I don’t care what you think, <he’ll have the resilience built up.>” (Father of a child affected by AI)*

## 4. Discussion

This qualitative study took a descriptive approach to explore the experiences and perspectives of both mothers and fathers of young children affected by AI and DI. Both mothers and fathers nowadays share parental duties in providing physical and emotional care to their children, though to a variable extent in different families. Nevertheless, fathers are seldom included in parental perception studies and such studies were dominated by mothers [20,21,22,23]. This study was the first study to include parents of young children affected with AI and DI and it explored father’s views alongside mothers’ views.

Parents were divided into two focus groups by gender, a group of fathers and another group for mothers intending to provide more comfortable group dynamics and aid the in depth exploration of the topic. Evidence from social research suggests that men and women interact differently in group situations and that they interact differently in mixed gender groups as opposed to same-gender groups. In comparison to same-gender groups, concerns about interpersonal relations in mixed-gender groups are greater and might limit the diversity of opinions expressed. Women in mixed-gender groups tend to be less dominant than in all female groups. Men might speak less about themselves in same-gender groups due to increased concerns about status and competition, however, they are generally less likely to conform to group pressure [24].

In this study, mothers and fathers highlighted the emotional and psychosocial challenges experienced by families from the time of tooth eruption. Both mothers and fathers expressed concerns about their children feeling different and shared similar concerns in relation to the impact of these dental anomalies on their affected children. This study agrees with the contemporary literature demonstrating that dentofacial aesthetics is considered important during childhood starting from a very early age and without any gender difference [5,25,26]. This study revealed parents’ beliefs on an increased worldwide media driven social expectation of white well aligned teeth that extends to affect young children which correlates to previous reports in adolescents and adults [3,27,28,29].

In this study, parents reported that their children’s self-perception of “being different” started to develop in conjunction with the eruption of primary teeth and before or approaching three years of age. This finding supports the growing evidence from children’s developmental psychology that self-perceptions starts to develop as early as two to three years of age [26,30,31,32]. 

It must be acknowledged, however, that the findings reported here are based on parents’ perspectives which might be distinct from the child’s own reports, especially in terms of emotional and social wellbeing of affected children [33,34,35]. In a qualitative study involving AI affected older children (10–16 years), the majority of children self-reported that they started to notice the altered dental appearance around six years old [15]. Another qualitative study of children (10–15 years) reported that the transition to secondary school was the age at which developmental enamel defects became a concern [8]. Children involved in these studies were older than affected children in our study. Younger children may lack the linguistic and cognitive skills to communicate their thoughts and perceptions which might explain the lack of child’s self-reported studies for younger age groups. In addition, child’s perceptions are known to change over time as part of the social, emotional, cognitive, and language development [33].

Both mothers and fathers in our study believed there is an increased emphasis on appearance as children approach school age due to increased social interactions and comparison with peers. It was apparent from focus group transcripts of these parents that young children at preschool and primary school age could notice affected teeth and can judge and react negatively to affected individuals similarly reported by Soares [36]. Worries about bullying and its impact on the child’s wellbeing from tooth eruption led parents to seek early dental care in an attempt to help their children. Such treatment was viewed as a way of achieving normality and “protecting children” from feeling different and/or experiencing negative social reactions, thus facilitating child’s social inclusion. No parent regretted their decision to obtain dental treatment for their child. These findings mirror those in the cleft lip and palate literature for parents of children from infancy to early adulthood [37], however, was never previously reported for parents of young children with dental anomalies.

The results of this study indicated that the personal experience of parents affected by AI/DI plays a pivotal role in parent’s judgements of their children’s teeth and perceived need for dental treatment. Affected parents who suffered from sensitivity limited their children’s consumption of cold drinks and ice cream as a precaution against sensitivity and, thus, might explain the less prominent mention of sensitivity issues experienced by children. Fear of bullying, as well as the childhood experience with bullying of affected parents, motivated them to seek early dental interventions. In addition, affected parents who had positive dental experience showed enthusiastic attitude toward dental treatment while negative dental treatment experience of one affected parent hindered him seeking early dental interventions for his child. This is a novel finding that was not previously reported in the AI and DI literature.

Although the main themes emerged from both focus groups were similar, certain concepts were more prominent in one group over another. While, the feeling of guilt was more prominent in the fathers’ focus group, the emotional stress and guilt associated with treatment under general anaesthesia were more prominent in the mothers’ focus group. This might be attributed to a gender difference in parents’ feelings and/or their ability to interpret their emotional stress. To the best of our knowledge, this is the first study to report parental feelings of guilt due to inherited dental anomalies which appears more likely to affect fathers of young children. This feeling of guilt is distinct from feelings of guilt due to dental caries as both AI/DI are inherited and not related to oral health behaviour.

The psychosocial and aesthetic impact of early dental intervention were more frequently described in our study compared to the functional impacts. Parents believed that early dental interventions precluded child’s “feeling different” or being seen or treated differently. An important and previously undescribed outcome for those who had early partial/full mouth restorative treatment was that they did not notice that their teeth were different until getting their permanent teeth. Another novel positive outcome after dental treatment of children’s primary teeth affected by hypoplastic AI, was the satisfaction with smoothness. This outcome was highly valuable for these children.

Whether affected by same condition or not, parents appeared to rely solely on the professional paediatric dentist in making all treatment related decisions. This echoes the work of Nelson (2012) with parents of children with cleft lip and palate [37,38]. Placing the trust in the specialist can be viewed as a way to resolving their feelings of anxiety in relation to their children’s treatment by constructing a sense of “being in the right hands”. Parents relied on the paediatric dentist to obtain information and advice on the short term and long term needs of their children. Professional opinion on decisions between different types of treatment and how it should be provided was essential. Hastings et al. previously proposed that dentist has the greatest influence on parents’ decision to use general anaesthesia [39]. This places the responsibility on the paediatric dentist to understand the parents’ own motivations for treatment, social considerations and the affected parents’ experiences on an individual basis, provide adequate information (based on the best available evidence) to enable parents to provide truly informed consent for the dental care of their child and, most importantly, act on the best interest of both child and family. It is important to note that all treatment provided was free of charge, so financial considerations were not a barrier to care for these children or parents.

An important outcome of this study was that focus group meetings gave parents participating in the study the opportunity to meet parents of children affected by same condition for the first time in their life. Parents were happy to share their emotions and experiences with other parents and felt belonging to a community, however, they espoused the need for additional information and emotional support. Parents reported a lack of electronic information relevant to their child and written in layman terms. Using a website or electronic professional led- support groups was one way suggested by parents as a source for both emotional and informational support, especially for newly diagnosed parents when there is no family history. Social peer groups for both children and parents were also suggested to get emotional and social support for children and their parents. This need was previously identified in a qualitative study of adolescents affected by AI and their parents where parents expressed their need for support groups held by professionals [40]. Peer support provides parents a sense of normality, practical advice, experiential learning and allows them to see into the future particularly when dealing with parents of older age [41]. Dentists should participate in the foundation of a website or electronic support group to ensure parents and families of affected children are well supported emotionally and with appropriate information.

An acknowledged limitation of this study was that parents were identified from existing patients with dental anomalies that were referred to the DDUH for dental assessment and treatment and so might have additional concerns that not necessarily reflect those with parents who did not seek referral for their AI and DI affected children. Another potential limitation is that all participants were Irish, all were married, and none were identified as low socioeconomic class, therefore, these results must be taken with caution as parents’ perspectives might vary for other ethnic groups, different socioeconomic classes, and/or single parents. It would be interesting to utilise the same research methodology to elicit parents’ views in different cultures and different dental settings where parents need to pay for treatment.

## 5. Conclusions

- Parents of children affected with AI and DI believe that “baby teeth are important” and value dental treatment of their children’s primary teeth before starting school.
- The personal experience of parents affected with AI/DI plays a pivotal role in parent’s judgements of their children’s teeth and perceived need for dental treatment.
- The paediatric dentist has a powerful influence on parental decisions and must acknowledge the parents perspectives when discussing options for treatment.
- Finance should not be a barrier to parents and children seeking care for AI/DI.

## Figures and Tables

**Table 1:** Demographic characteristics of fathers’ focus group.

Participant	Age	Affected by the Same Condition	Child Affected by AI/DI	Number, Gender (Age) of Affected Children	Dental Intervention in Primary Dentition Stage
1	≥35	No	AI	1 Male (Age 6)	Yes
2	≥35	No	DI	1 Female (Age 10)	Yes
3	≥35	No	DI	2 Female (Age 2) Male (Age 6)	Yes
4	≥35	Yes	AI	1 Male (Age 9)	No
5	<35	Yes	AI	2 Male (Age 6) Female (Age 2)	Yes
6	≥35	Yes	AI	1 Male (Age 9)	Yes

**Table 2:** Demographic characteristics of mothers’ focus group.

Participant	Age	Affected by the Same Condition	Child Affected by AI/DI	Number, Gender (Age) of Affected Children	Dental Intervention in Primary Dentition Stage
1	≥35	No	DI	3 Males (Age 16, 13 and 10)	Yes
2	≥35	No	AI	1 Male (Age 9)	No
3	≥35	Yes	AI	1 Male (Age 6)	Yes
4	≥35	No	DI	1 Female (Age 7)	Yes
5	<35	No	AI	2 Male (Age 6) Female (Age 2)	Yes
6	≥35	No	AI	2 Male (Age 7) Female (Age 10)	Yes
7	≥35	Yes	DI	2 Male (Age 6) Female (Age 2)	Yes
